# Supplementary material for: Effects of Artificial Light at Night (ALAN) on European Hedgehog Activity at Supplementary Feeding Stations
Source: Animals (Basel). 2020 Apr 28;10(5):768. doi: 10.3390/ani10050768 (PMC7278375; doi:10.3390/ani10050768)
Supplement: Supplementary file 1 [file animals-10-00768-s001.pdf]

Supplementary material

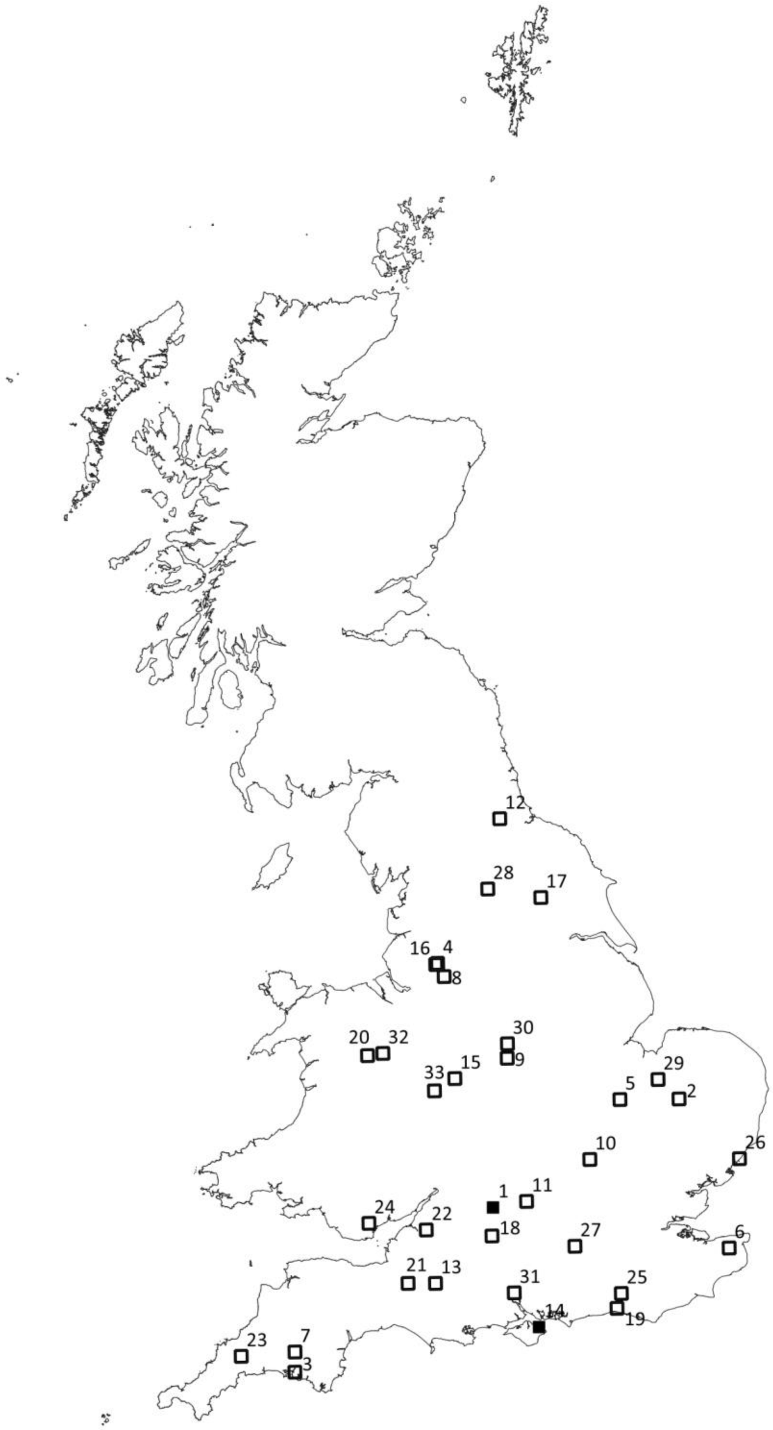

Figure S1. The spatial distribution of the 33 project volunteers. The 31 points in white were included in the activity pattern analyses, whereas the two in black were not due to insufficient sample sizes for activity analyses.

Table S1. Site-level results of activity pattern analyses giving the sample size (hedgehog records) in both the dark and light treatments, the coefficient of overlap between the two activity patterns (Δ), and the p-value test statistic of the Wald test performed. If $p < 0.05$ (denoted with *) then the activity patterns are significantly different between treatments. Activity analyses were not conducted for sites 1 and 14 due to sample sizes of less than 10.

Site	Dark sightings (n)	Light sightings (n)	Overlap (Δ)	p-value
1	1	4	NA	NA
2	53	24	0.52	0.00*
3	200	103	0.90	0.332
4	66	35	0.93	0.779
5	49	79	0.81	0.07
6	69	67	0.84	0.068
7	36	26	0.73	0.007*
8	15	16	0.75	0.213
9	77	79	0.85	0.083
10	52	70	0.80	0.034*
11	124	101	0.77	0.001*
12	108	132	0.87	0.119
13	29	53	0.68	0.009*
14	8	2	NA	NA
15	18	15	0.77	0.248
16	95	73	0.75	0.00*
17	52	46	0.92	0.804
18	55	234	0.67	0.00*
19	38	26	0.92	0.812
20	41	42	0.89	0.427
21	13	19	0.85	0.536
22	22	18	0.90	0.686
23	30	26	0.70	0.023*
24	61	48	0.74	0.017*
25	131	67	0.88	0.203
26	101	87	0.74	0.002*
27	104	161	0.93	0.629
28	268	187	0.86	0.001*

29	122	193	0.77	0.00*
30	57	71	0.89	0.367
31	124	70	0.95	0.884
32	57	79	0.88	0.461
33	80	87	0.64	0.00*

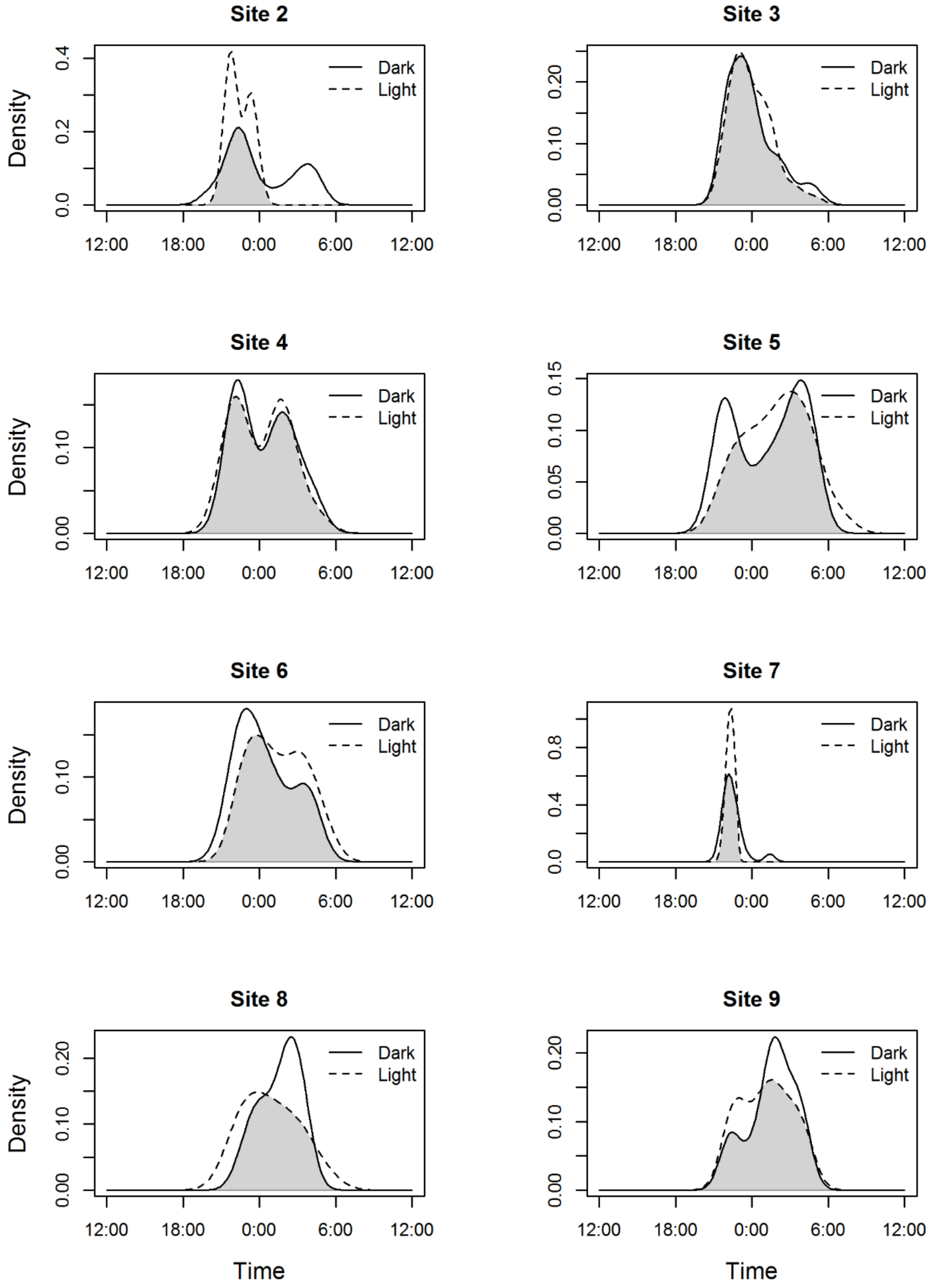

Site 2
Site 3
Site 4
Site 5
Site 6
Site 7
Site 8
Site 9
Density
Time
Dark
Light
12:00
18:00
0:00
6:00
12:00

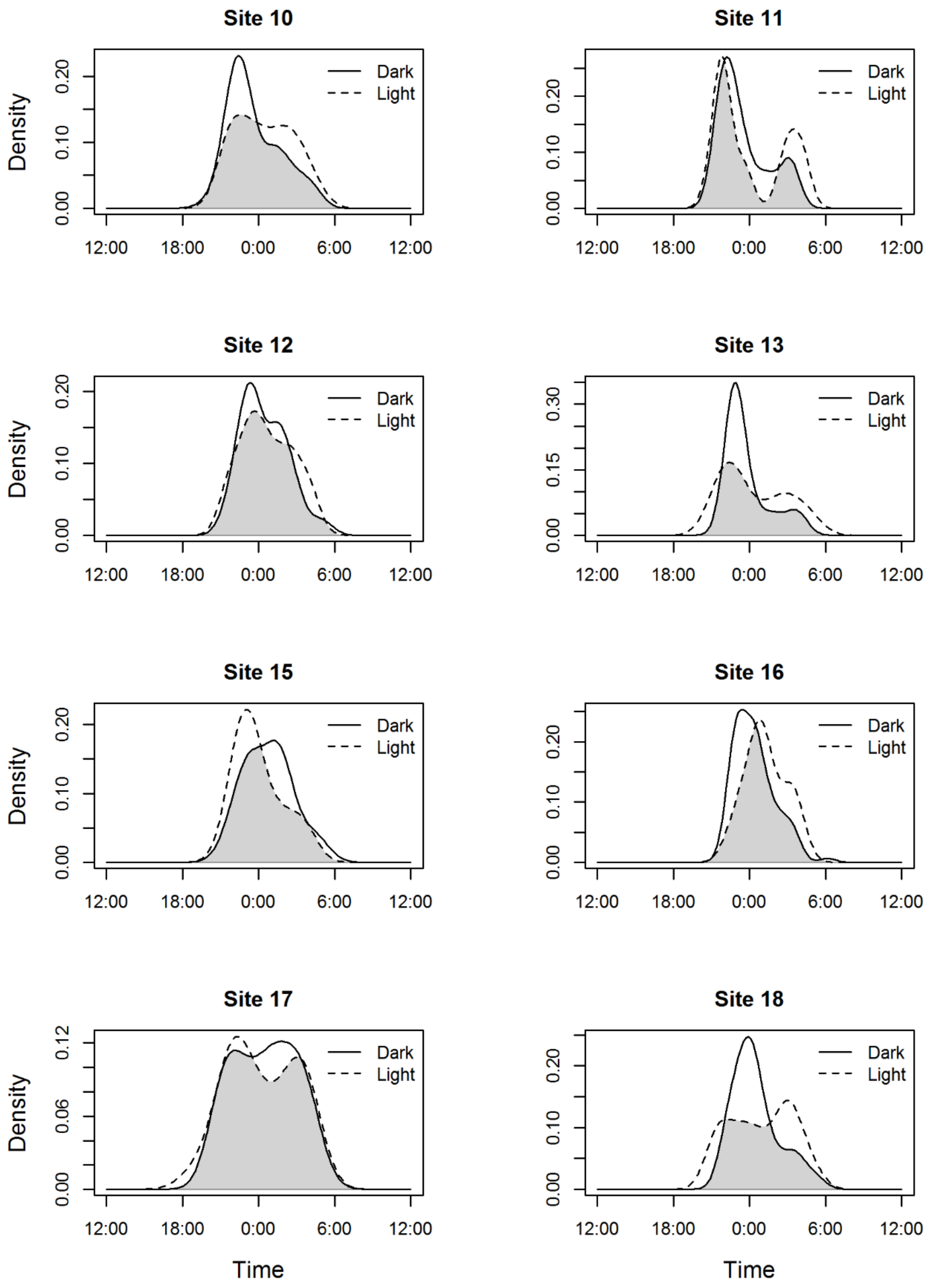
Site 10
Site 11
Site 12
Site 13
Site 15
Site 16
Site 17
Site 18
Density
Dark
Light
0.00
0.10
0.20
0.15
0.30
0.06
0.12
12:00
18:00
0:00
6:00
12:00
Time

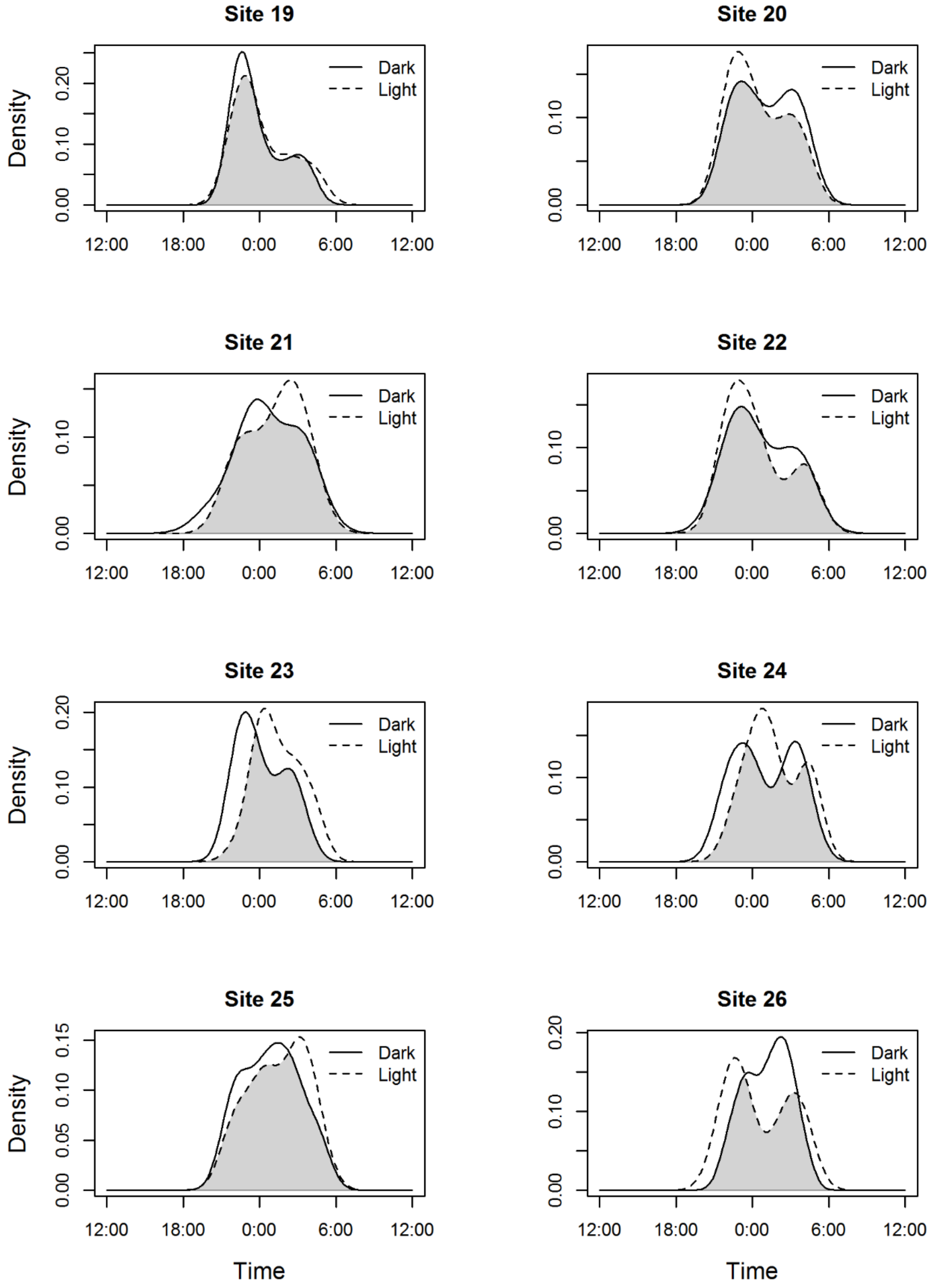
Site 19
Site 20
Site 21
Site 22
Site 23
Site 24
Site 25
Site 26
Dark
Light
Density
Time
0.00
0.10
0.20
0.05
0.15
12:00
18:00
0:00
6:00
12:00

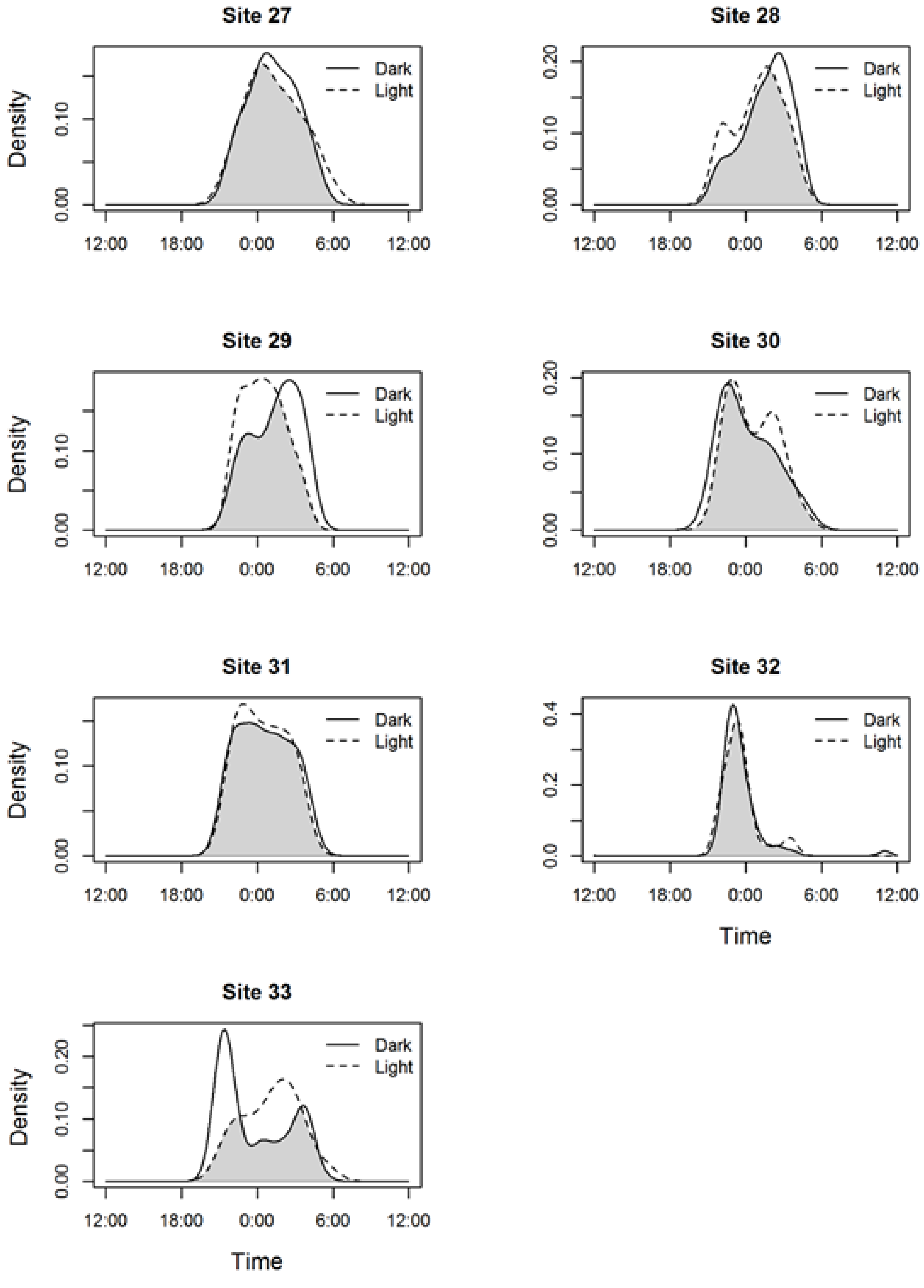

Figure S2. Activity patterns of hedgehogs, expressed as a kernel density, at each of the 31 sites in the week where the light was on (dashed line) and when the light was off (solid line). The area shaded in grey is where the two activity patterns overlap (Δ). Sites 1 and 14 were removed due to small sample sizes (n < 10).
